# Electrochemical detection of kynurenic acid in the presence of tryptophan with the carbon paste electrode modified with the flower-like nanostructures of zinc oxide doped with terbium

**DOI:** 10.3389/fchem.2023.1250994

**Published:** 2023-09-22

**Authors:** Ali Amir Poursaeed, Shohreh Jahani, Mehran Moradalizadeh, Mehdi Shahidi Zandi, Mohammad Mehdi Foroughi

**Affiliations:** ^1^ Department of Chemistry, Kerman Branch, Islamic Azad University, Kerman, Iran; ^2^ Noncommunicable Diseases Research Center, Bam University of Medical Sciences, Bam, Iran

**Keywords:** kynurenic acid, tryptophan, carbon paste, zinc oxide, terbium

## Abstract

With the help of a hydrothermal approach in this study, we could provide flower-like nanostructures (NSs) of zinc oxide (ZnO) doped with Tb (FL-NS Tb^3+^/ZnO). Then, FL-NS Tb^3+^/ZnO morphology was investigated by energy-dispersive X-ray spectroscopy (EDX), scanning electron microscopy (SEM), X-ray powder diffraction (XRD), and map analysis. The results revealed higher activity centers and porosity of this nanocomposite, which were followed by acceptable electrochemical function. Hence, it can be utilized for fabricating an electrochemical sensor with an appropriate response for the simultaneous determination of kynurenic acid (KYN) and tryptophan (TRP). However, as compared with the modified carbon paste electrode (FL-NS Tb^3+^/ZnO/CPE), the bare carbon paste electrode (BCPE) exhibited a weak response toward KYN and TRP but the modified electrode was followed by a high current response for KYN and TRP at a potential 0.35 and 0.809 V. Therefore, cyclic voltammetry (CV) was applied in optimal experimental conditions to study the electrochemical behaviors of KYN and TRP over the surface of the proposed modified electrode. Moreover, we used differential pulse voltammetry (DPV) for quantitative measurements. It was found that this new modified electrode linearly ranged from 0.001 to 700.0 μM, with detection limits of 0.34 nM and 0.22 nM for KYN and TRP, respectively. In addition, KYN and TRP in real samples can be analyzed by this sensor, with a recovery of 97.75%−103.6% for the spiked KYN and TRP in real samples.

## 1 Introduction

According to studies, tryptophan (TRP) is one of the major 20 essential amino acids provided by a diet. Unlike the other existing amino acids, TRP circulates in plasma and blood, generally attached to albumin. In fact, 10%–20% of this amino acid occurs as free form in the plasma (Pardridge, 1979; Floc’H et al., 2011). Its metabolization occurs in mammals through various pathways, and one of the basic pathways is kynurenic acid (KYN) in the central and peripheral systems. Researchers have also observed the involvement of enzymes catalyzing reactions in KYN in several disorders and illnesses, wherein an imbalance in the level of KYN and TRP has been shown. Moreover, TRP degradation via cytokine-induced indoleamine 2,3-dioxygenase (IDO) to formyl kynurenine increased in the case of the activation of the cellular immune system (Vazquez et al., 2001; Ruddick et al., 2006). KYN synthesis is catalyzed by kynurenine aminotransferase (KAT) which is one of the endogenous antagonists at the alpha 7 nicotinic cholinergic receptors and glycine site of N-methyl-d-aspartate (NMDA), with a fundamentally neuroactive metabolite whose damage is linked to numerous acute disorders and illnesses in the nervous system (Rossi et al., 2008). In addition, KYN contributes to hypertension or high blood pressure and diabetes pathogenesis, and controlling its activities is of high significance. Thus, measuring KYN and TRP in plasma has been attractive for timely diagnoses of the respective illnesses (Andrzejewska-Buczko et al., 2001; Zhao et al., 2010).

Scholars have presented numerous HPLC methods for determining KYN and TRP in biological samples. In general, fluorescence detection has been applied for measuring TRP and KYN and other approaches such as coulometric detection and capillary electrophoresis with laser-induced fluorescence determination have been employed for detecting KYN and TRP; however, various matrices influence their usability (Hansen and Lunte, 1997; Maneglier et al., 2004; Kawai et al., 2007; Ma et al., 2009; Soto et al., 2011; Van et al., 2014). Researchers mentioned disadvantages such as laborious analysis, costly processes, complications, exposure to diverse interferences, derivatization reactions, and pollution (Maaref et al., 2018; Farvardin et al., 2020; Zheng et al., 2020; Jahani et al., 2022; Tang et al., 2022; Zhang et al., 2023; Ren et al., 2023; Özcan et al., 2023). It was demonstrated that electrochemical approaches are efficient analytical procedures with the following characteristics: easy to use, rapid response, inexpensiveness, and portability (Akbari et al., 2020; Iranmanesh et al., 2020; Moarefdoust et al., 2021; Antherjanam and Saraswathyamma, 2022; Zhang et al., 2023; Cai et al., 2023; Tang et al., 2023; Wan et al., 2023). In fact, scholars have increased the sensitivity for detecting the electroactive analytes using chemical modifiers and a wide range of nanomaterials have been applied to determine the analytes (Wu et al., 2023; Kambale and Lokhande, 2023; Zhao et al., 2023; Taherizadeh et al., 2023; Guo et al., 2023; Ghasemi et al., 2023).

As mentioned in the above paragraphs, ZnO has been realized as one of the semiconductors with the most acceptable electrochemical and optical features. Various morphologies, including wires, rods, sheets, flowers, and tubes can be used to synthesize nanosized ZnO (Song et al., 2019). Research has shown that the greater specific surface area of ZnO nanoflowers with a three-dimensional (3D) structure is appropriate for very good electrochemical functions and ion exchange (Zhang et al., 2020). Even though nanosized ZnO is experiencing improvement, it suffers from some limitations in the electrochemical sensors, which is caused by its lower electrical conductivity. Doping with rare earth metals has been shown to be a main approach for improving the electrical conductivity of ZnO (Sharma and Sahay, 2023).

For the first time, we have synthesized and characterized an effective material, flower-like nanostructures (NSs) of ZnO doped with Tb (FL-NS Tb^3+^/ZnO), to modify carbon paste electrodes (CPEs). In addition, a literature review showed no studies on the electroanalysis and simultaneous detection of kynurenic acid (KYN) and tryptophan (TRP) exploiting modified electrodes with novel nanocomposites. As seen in the related steps for the modified electrode, FL-NS Tb^3+^/ZnO exhibited a greater conductivity and active area, with faster electron transport over the electrode surface, which promoted a response of the CPE signal. Moreover, differential pulse voltammetry (DPV), electrochemical impedance spectroscopy (EIS), and cyclic voltammetry (CV) were chosen to monitor the modification process of the electrode and DPV and CV were employed to examine KYN and TRP electro-oxidation over the FL-NS Tb^3+^/ZnO/CPE surface. Finally, we studied the function of FL-NS Tb^3+^/ZnO/CPE in terms of the simultaneous detection of both analytes.

## 2 Experimental design

### 2.1 Instruments

In this stage, we utilized an electroanalyzer made by SAMA 500, Isfahan, Iran for chronoamperometric and voltammetric measurements and EIS. This 3-electrode cell was made up of a platinum wire as the counter electrode, Ag/AgCl (saturated KCl) as the reference electrode, and a carbon paste electrode as a working electrode. Moreover, X-ray powder diffraction (XRD) data were gathered using a Philips PC-APD X-ray diffractometer made in the Netherlands and the modifier was described by energy dispersive spectroscopy (EDS) and scanning electron analysis (EM 3200 SEM and KYKY, provided from China). Finally, we used a digital pH meter (ELICO LI 120) for the pH value measurement.

### 2.2 Chemicals

Graphite powder, ethylene glycol, zinc nitrate, sodium dihydrogen phosphate, disodium hydrogen phosphate, paraffin oil, terbium chloride, sodium hydroxide, *polyvinylpyrrolidone*, and phosphoric acid, made by Merck Co., were the chemicals chosen for this research. It should be noted that each chemical had high analytical purity (≥99% with the exception of phosphoric acid with 85% purity). We applied double-distilled water to prepare the solution.

### 2.3 Cells and electrochemical detection

Electrochemical measurements were done using a 3-electrode cell in a computer-connected electrochemical system, which included a platinum wire as the auxiliary electrode, unmodified and modified CPEs as the working electrodes, and an Ag/AgCl electrode as the reference electrode.

### 2.4 Preparation of the solution

As mentioned above, we employed double-distilled water in each stage of the experiments for washing dishes and preparation of the aqueous solution. Then, the weighted KYN and TRP were added to fresh distilled water in volumetric flasks to prepare various concentrations of KYN and TRP. Furthermore, we prepared fresh buffer and drug solutions daily and a specific concentration of the above materials was diluted using a buffer solution at a given pH to prepare the sample solution of the blood serum, and the standard addition method was utilized to transfer a certain content to the electrochemical cells for voltammetric measurements. Then, we initially cleaned the whole container with a solution of nitric acid and water (1: 1), and the glassware was washed with deionized and distilled water.

### 2.5 Synthesis of the flower-like NSs of ZnO doped with Tb

To provide the flower-like NSs of ZnO doped with Tb, 0.1 g of terbium chloride and 2 g of zinc nitrate and PVP were dissolved in 100 mL of ethylene glycol. After stirring, we transported the obtained mixture to an autoclave at 200°C for 12 h. In the next step, filtration of the precipitate and washing with ethanol and water were performed 3 times. The calcination of the precipitate was done at 500°C for 4 h, and then it was completely dried at 80°C for a 12 h period.

### 2.6 Electrode preparation

#### 2.6.1 CPE

In this step, we mixed graphite powder and paraffin oil at the ratio of 30:70 using a CPE and squeezed the paste into an insulin syringe with an aperture diameter of 2 mm. Then, for refreshing the electrode surface, a syringe piston was applied to eliminate 1 mm of the paste, and polishing was performed on a clean piece of paper through vertical abrasion. After that, distilled water was used for washing its surface for additional measurements and a thin copper wire was inserted in the CPE from the syringe end to establish an electrical link between the electroanalyzer and the obtained working electrode.

#### 2.6.2 CPE modified with the NSs

For this step, we utilized a glass and a balance to weigh 0.5 g of graphite, added it to a mortar, and powdered it for 20 min. Then, 0.004 g of the FL-NS Tb^3+^/ZnO was added and mixing was done for an additional 20 min to achieve the desired paste. Then, 7 drops of paraffin were poured into it to make this paste more homogeneous and softer. Finally, the electrodes were labeled.

### 2.7 Measurement of the real samples of KYN and TRP

Upon the preparation of the blood serum or urine samples, we poured 0.9 mL of 15 w/v % solution of Zn sulfate/acetonitrile into 1 mL of the sample of human plasma or urine and kept the test tube at 40°C for 15 min. Furthermore, solution centrifugation was performed to settle the proteins to reach a fully transparent blood serum or urine sample. Afterward, the buffer was poured for a 5-fold dilution of the blood serum or urine sample, and various amounts of KYN, TRP, and standard solution were poured into the final diluted blood serum. Finally, the DPVs were shown, and the percentage of the recovery of KYN and TRP was specified using the standard addition approach.

### 2.8 Electrochemical approach

Cyclic voltammetry (CV), chronoamperometry (CHA), and differential pulse voltammetry (DPV) were applied for the electrochemical study and quantification of KYN and TRP, respectively.

CV was performed in a phosphate buffer (0.1 M, pH 7.0) with and without the presence of KYN and TRP, starting with the equilibrium potential in the anodic direction using a potential window of −0.5–1.2 V at different scan rates. Anodic peaks were analyzed in order to establish the relationship between the maximum current intensity of the anodic peaks and the scan rate.

Under optimized conditions, CHA experiments were carried out at an applied potential of 0.40 and 0.86 V versus SCE using different concentrations of KYN and TRP, respectively.

In order to achieve the higher analytical response (anodic current), the optimal conditions for DPV measurements were as follows: PBS, pH of 7.0, modulation amplitude of 0.02505 V, modulation time of 30 ms, interval time of 200 ms, step potential of 10 mV, initial potential = 100 mV, and end potential of 950 mV. To achieve the DP voltammograms of KYN and TRP, appropriate volumes of the stock solutions of drugs were added to the cell containing supporting electrolytes to a total volume of 25 mL.

## 3 Results and discussion

### 3.1 Structural examination

**FIGURE 1:**
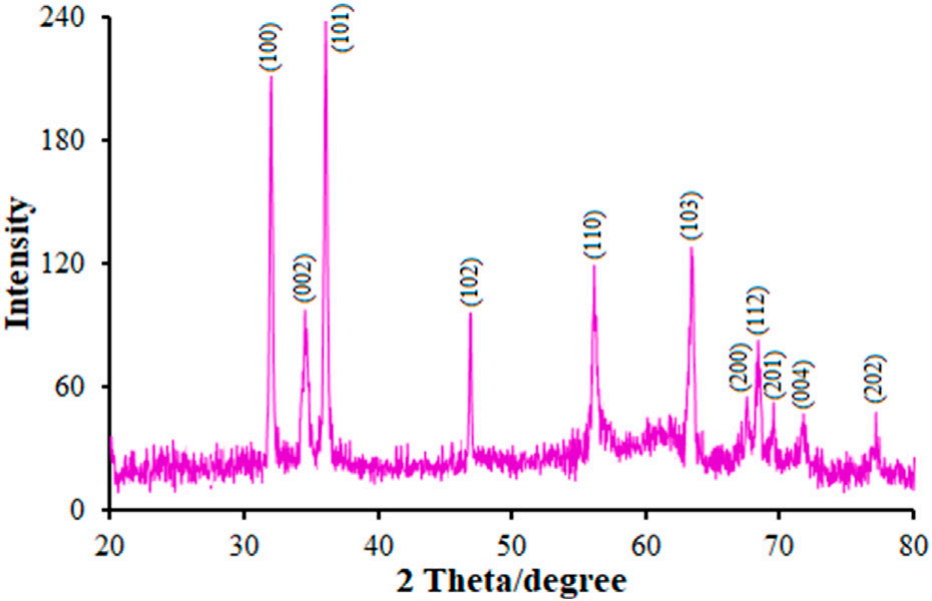
XRD pattern of FL-NS Tb^3+^/ZnO.

The XRD in Figure 1 shows the crystalline nature of the nanoflowers in that a 2θ peak was observed at 32.56° (100), 34.83° (002), 36.415° (101), 47.14° (102), 56.22° (110), 63.59° (103), 67.75° (200), 68.63° (112), 69.73° (201), 71.70° (004), and 77.22° (202); however, we did not observe any characteristic peak for other impurities such as Zn (OH)_2_ and metallic Zn, reflecting the product purity. There was a complete correlation between the peaks and polycrystalline hexagonal wurtzite-structured ZnO; that is, three pronounced peaks (100), (002), and (101) at 2θ = 32.56°, 34.83°, and 36.41° that could be compared to the typical XRD pattern of the standard ZnO (JCPDS 89–7,102) (SudapalliShimpi, 2023). It should be noted that ZnO NF had higher intensity and narrower peaks, resulting in greater crystallinity. In Figure 1, a clear shift of the diffraction peaks towards a higher angle than pure ZnO is shown, demonstrating the significantly greater ionic radius of Tb (237 p.m.) in comparison to Zn (139 nm) that may be due to the minor doping of Tb ions into the ZnO lattice, leading to an improvement in the lattice parameter in the Tb-doped ZnO crystallites. Researchers have estimated these minor changes in the Tb replacing Zn ions in the lattice without any variations in the crystal lattice (Taherizadeh et al., 2023).

**FIGURE 2:**
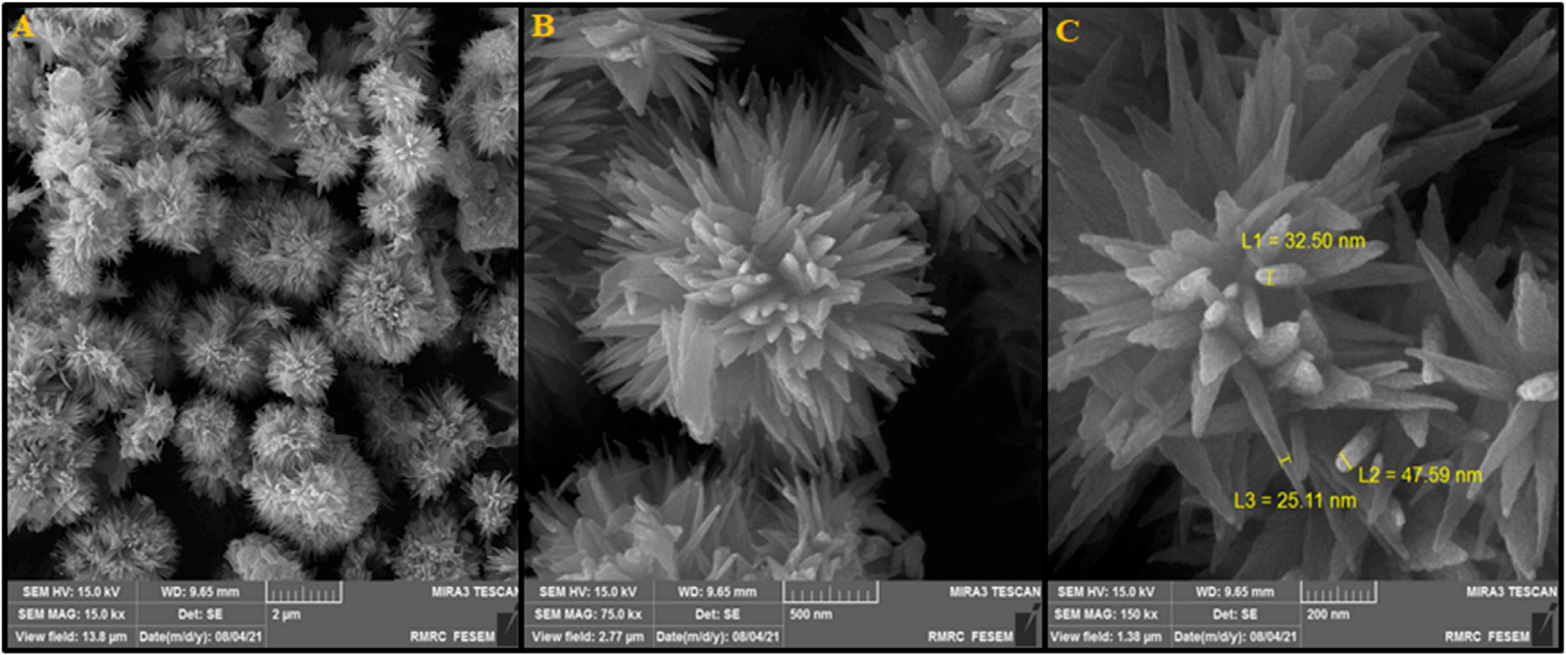
**(A)** FESEM image, **(B)** and **(C)** High resolution FESEM image of FL-NS Tb^3+^/ZnO.

In this step, FESEM was used for the characterization of the morphology of the as-synthesized Tb-doped ZnO nanoflowers. Figure 2A shows the flower-like 3D morphology of the ZnO nanoflakes formed in the petals in the FESEM images at a lower magnification. Consequently, high-density growth was observed because of the nanoflakes’ self-assembly. Moreover, Figures 2B, C shows FESEM images at high magnification, containing uniform nanoflowers. As shown in Figures 2A, C single nano-flower dimension was ∼1–3 µm with multiple nanoflakes with radial growth from the center in symmetry so that most of the nanoflowers were associated with each other.

**FIGURE 3:**
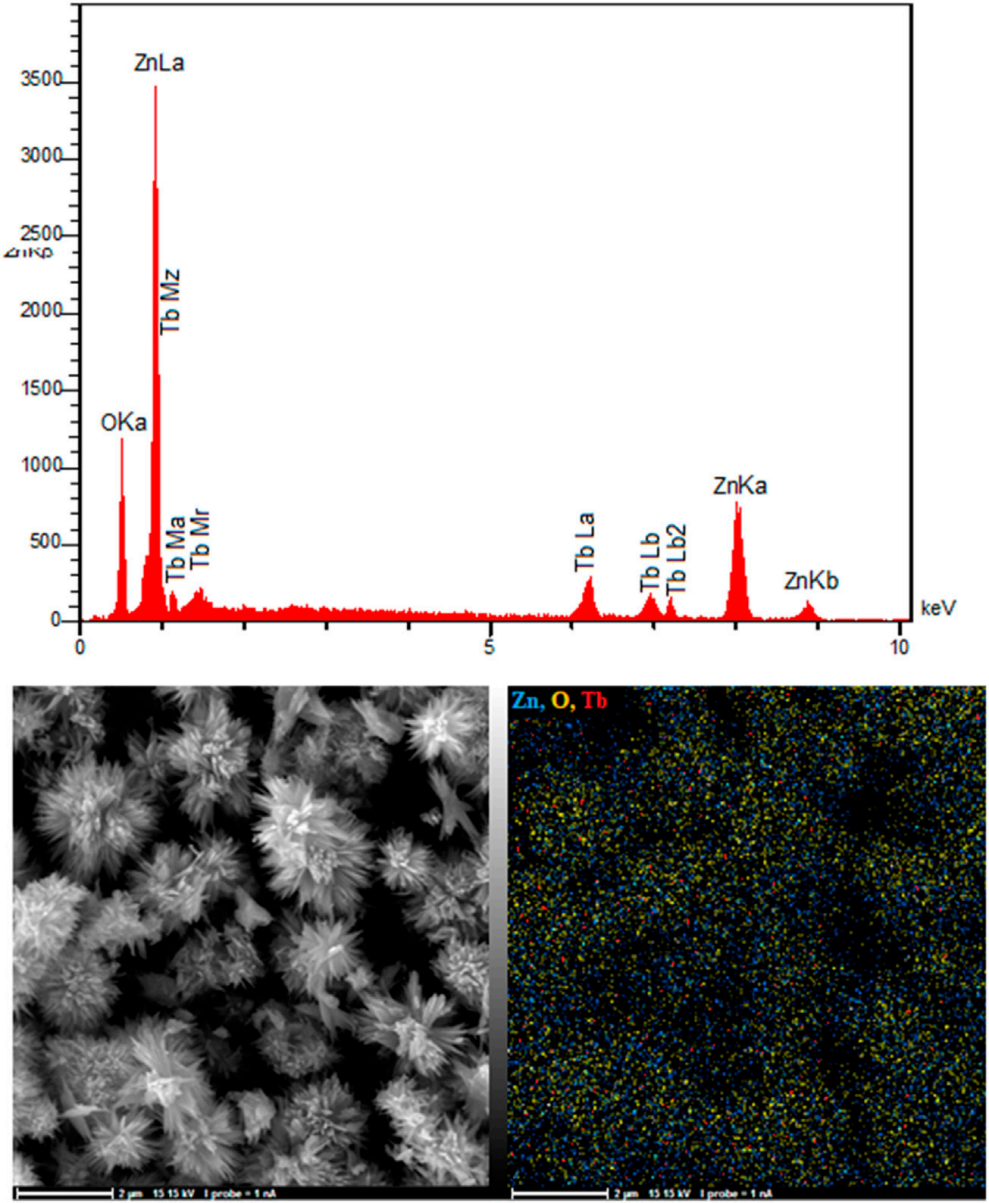
EDX spectra and elemental mapping of FL-NS Tb^3+^/ZnO.

EDAX analysis, shown in Figure 3, proved the presence of Tb, O, and Zn in the ZnO nanoflowers. As shown in Figure 3, Tb, O, and Zn had matching elemental mapping, with a smooth distribution of these three elements in the sample according to the results obtained from the elemental mapping.

### 3.2 Electrochemical behaviors of the proposed modified electrodes

**FIGURE 4:**
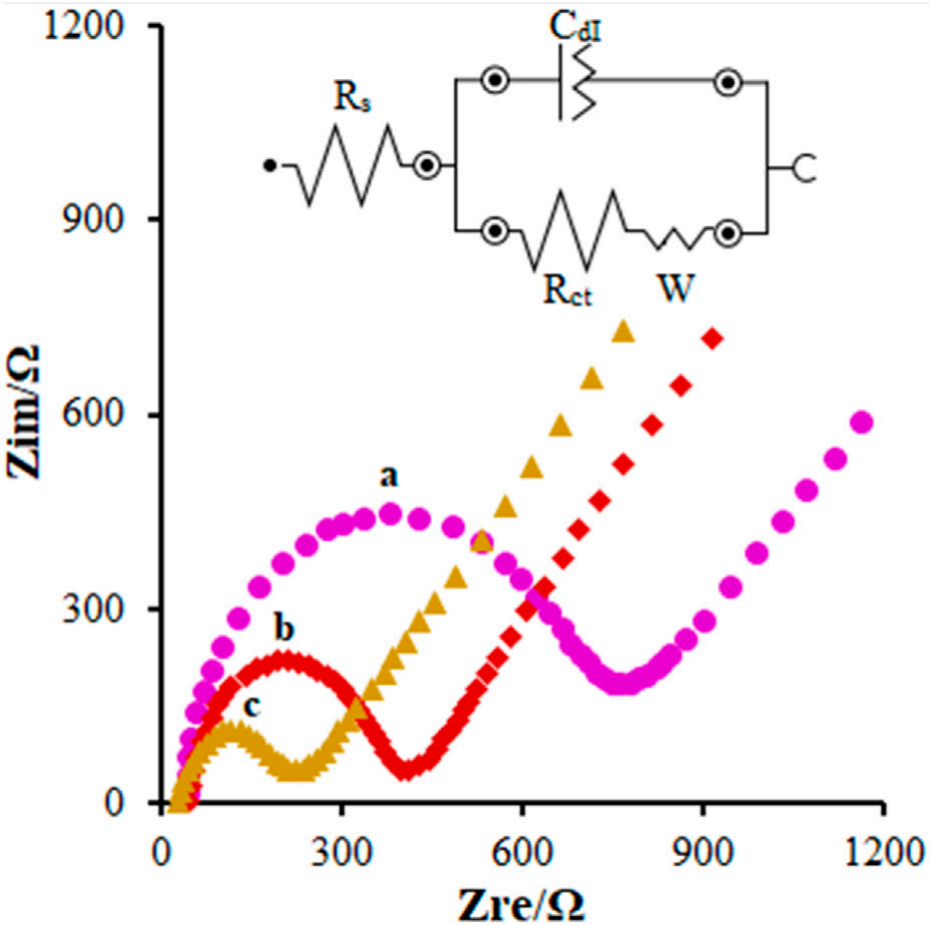
EIS diagrams and the equivalent circuit for 0.1 mM [Fe(CN)_6_]^3-^ solution at **(A)** bare CPE, **(B)** FL-NS ZnO/CPE, and **(C)** FL-NS Tb^3+^/ZnO/CPE in aqueous 0.1 M KCl. Frequency ranges from 100 KHz to 0.1 Hz.

According to our research design, EIS was applied to show the variations in the electrochemical behaviors of the modified electrode surface. Studies in the field have shown the ability of polymers, semiconductor materials, and nanomaterials coated on the surface of the electrode to change the transport resistance and double–layer capacitance of the corresponding electrode. Hence, this method could be used to achieve more data on the variations in the surface impedance of the electrodes in the course of the modification process of the electrode. The features of the electrochemical impedance of the modified FL-NS ZnO/CPE, FL-NS Tb^3+^/ZnO/CPE, and unmodified CPE in the solution with 1 mM of redox pair KCl 0.1 M and [Fe(CN)_6_]^3−^/[Fe(CN)_6_]^4−^ ranged between 0.1 Hz and 100 kHz, shown as the Nyquist curve (Z_re_ versus Z_im_) in Figure 4. The equivalent circuit was designed and implemented to understand and evaluate the individual components of the EIS system. The resistance of the solution (R_s_), double layer capacitance at the surface of the electrode (C_dI_), charge transfer resistance (R_ct_), and Warburg resistance (W) were simplified in the Randles equivalent circuits, as shown in Figure 4. Warburg resistance is the result of a diffusion process occurring at the electrode–electrolyte interface. As can be observed in the Nyquist diagram, the high resistance of the charge transfer for the unmodified CPE (Figure 4 (curve a)) resulted from the reduced transfer rate of the charge and mass on the surface of the electrode (792 Ω). Moreover, the electron transfer kinetics in FL-NS ZnO [Figure 4 (curve b)] were enhanced in the presence of the modifier in the electrodes and decreased the transport resistance of the charge (408 Ω). Additionally, the charge transfer was considerably enhanced around the modified electrode with the FL-NS ZnO mixture and Tb^3+^ (Figure 4 (curve c)) which could related to the higher conductivity of the modifier (218 Ω).

### 3.3 Specific surface area of the modified electrode

**FIGURE 5:**
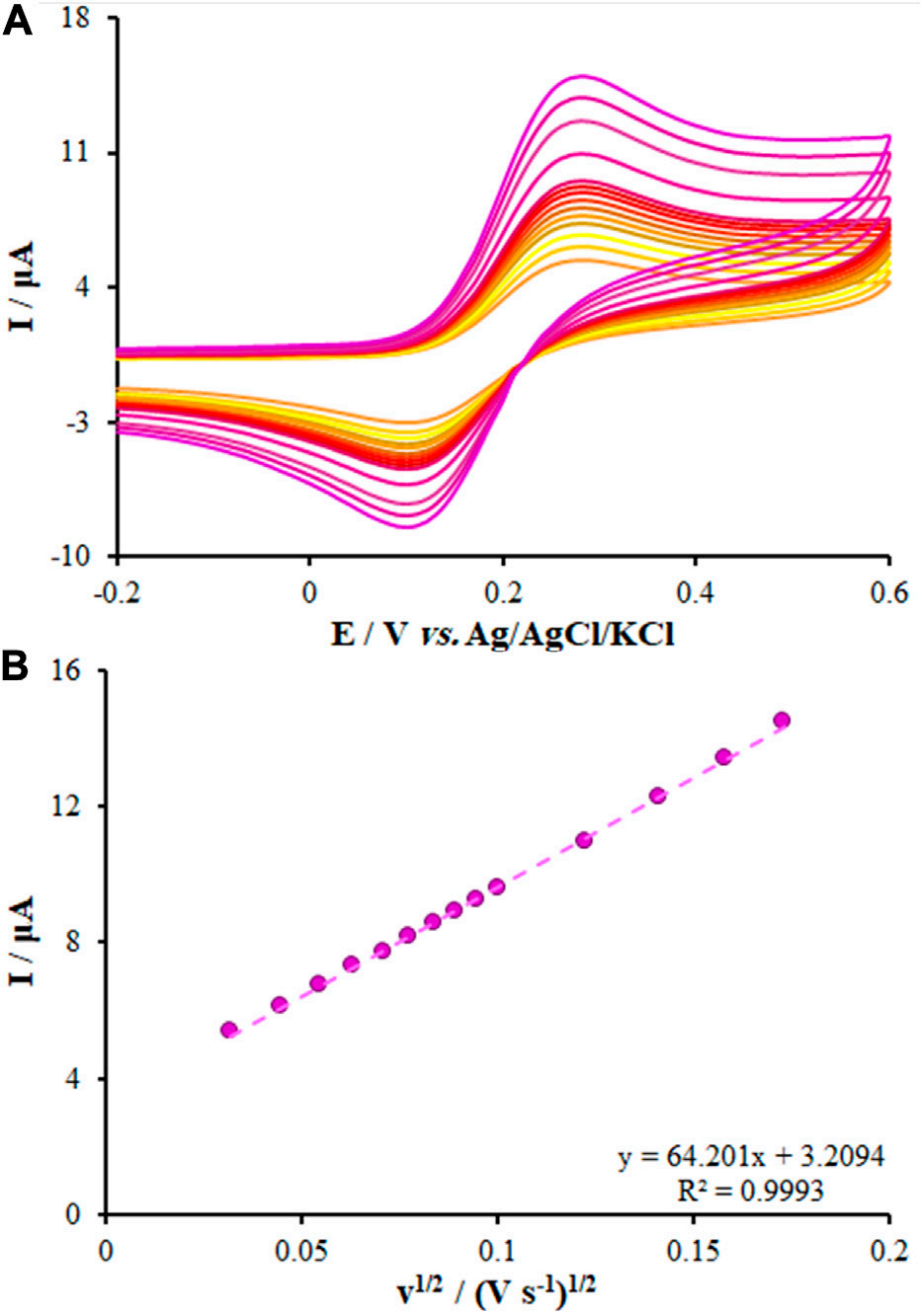
**(A)** CVs of FL-NS Tb^3+^/ZnO/CPE in the presence of 0.3 mM [Fe(CN)_6_]^3−^ solution in aqueous 0.1 M KCl at various scan rates (from inner to outer curve): 10, 20, 30, 40, 50, 60, 70, 80, 90, 100, 150, 200, 250, and 300 mV/s. **(B)** The plot of peak currents vs. υ^1/2^.

According to the research design, the Randles-Sevcik equation for a quasi-reversible electrochemical process (Eq. 1) and the CV were employed to calculate the effective surface area (in cm^2^) of electrode A. Hence, we used the CV of the soluble 0.3 mM of [Fe(CN)_6_]^−3/−4^ with the diffusion coefficient 7.6 × 10^−6^ (Bard and Faulkner, 2001):

$$I_{p}=\pm \left(2.63\times 10^{5}\right)\;n^{3/2}\;A\;D^{1/2}\;C\;v^{1/2}$$ (1)

where n stands for the number of electrons transported in the reduction and oxidation processes of [Fe(CN)_6_]^−3/−4^ (=1), D refers to the diffusion coefficient in cm^2^/s, and C represents the [Fe(CN)_6_]^−3/−4^ concentration (mol/cm^3^). The scanning rate and current are represented by V/s and A. From the slope of the plot of I_p_ vs. ν^1/2^, the surface area of the unmodified CPE was found to be 0.112 cm^2^ and for the FL-NS ZnO/CPE and FL-NS Tb^3+^/ZnO/CPE, the calculated surface areas were 0.228 cm^2^ and 0.301 cm^2^ (Figure 5; Table 1). The surface area of the FL-NS Tb^3+^/ZnO/CPE was more significant, which can be attributed to the presence of Tb^3+^ and FL-NP ZnO that led to high electrical conductivity and the specific surface area of the modified electrode.

**TABLE 1:** Surface area of the electrodes.

Electrode	Slope	Area (A, cm^2^)
CPE	24.01	0.112
FL-NS ZnO/CPE	48.69	0.228
FL-NS Tb^3+^/ZnO/CPE	64.20	0.301

### 3.4 Electrochemical behaviors of KYN and TRP over the modified and unmodified electrode surface

**FIGURE 6:**
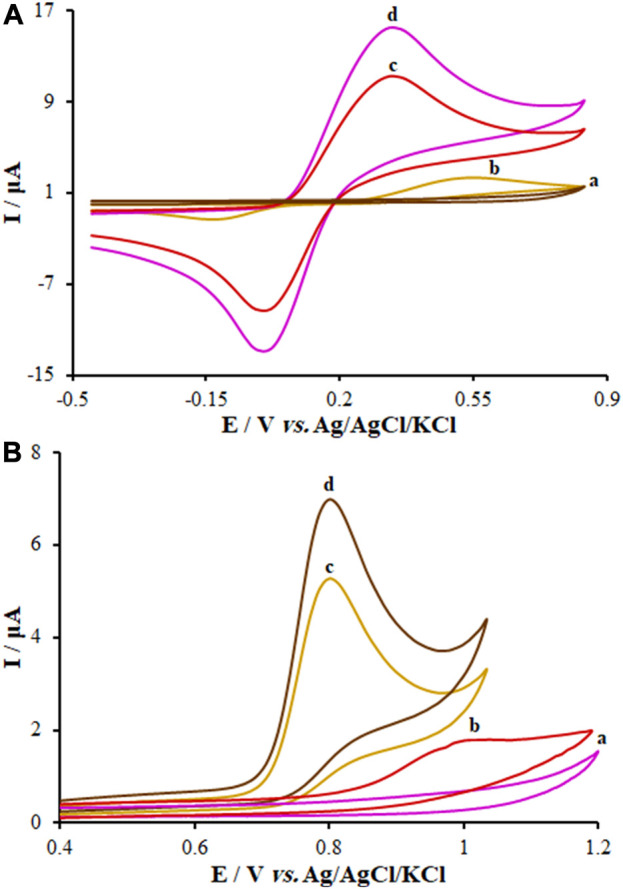
CVs of **(A)** FL-NS Tb^3+^/ZnO/CPE in the absence of analyte, **(B)** bare CPE, **(C)** FL-NS ZnO/CPE, and **(D)** FL-NS Tb^3+^/ZnO/CPE in presence of **(A)** KYN (250.0 µM) and **(B)** TRP (150.0 µM) in 0.1 M PBS (pH 7.0). In all cases, the scan rate was 50 mV s^−1^.

For this step, CV was applied to assess the electrochemical behaviors of KYN and TRP over the surface of the unmodified CPE, FL-NS Tb^3+^/ZnO/CPE, and FL-NS ZnO/CPE in 0.1 M PBS with a pH of 7.0 (Figure 6). Moreover, a solution of KYN (250.0 µM) and TRP (150.0 µM) in the PBS was added to the electrochemical cells, separately. See Figure 6 for the oxidation peak current of KYN and TRP with the peak oxidation potential of the solution over the electrodes’ surfaces. As seen in Figure 6, these voltammograms exhibited an oxidation peak for KYN and TRP at 0.54 V and 1.001 V for the unmodified CPE, which shifted to 0.350 V and 0.809 V for FL-NS Tb^3+^/ZnO/CPE, respectively. Different currents were observed for the various electrodes. The currents for the FL-NP Tb^3+^/ZnO/CPE and FL-NP ZnO/CPE were 6.7 and 4.86 times greater compared to the unmodified CPE. The results indicate the simultaneous effect of Tb^3+^ and FL-NP ZnO as modifiers in improving the KYN and TRP peak currents and thus increasing the sensitivity of the proposed method.

### 3.5 Optimization of the modifier amount

To achieve the best response in the electrochemical measurement of KYN and TRP, the amount of FL-NS Tb^3+^/ZnO used as a modifier in preparing carbon paste electrodes was optimized. For this purpose, electrodes with values of 0.002–0.006 g of FL-NS Tb^3+^/ZnO were prepared and placed in a 0.1 M phosphate buffer solution at a pH of 7.0 to measure 40.0 μM of KYN solution and 35.0 μM of TRP solution by DPV. The results indicated that 0.004 g of FL-NS Tb^3+^/ZnO has the highest current in the measurement of KYN and TRP.

### 3.6 Determination of the effects of the potential scan rate on the KYN and TRP oxidation

**FIGURE 7:**
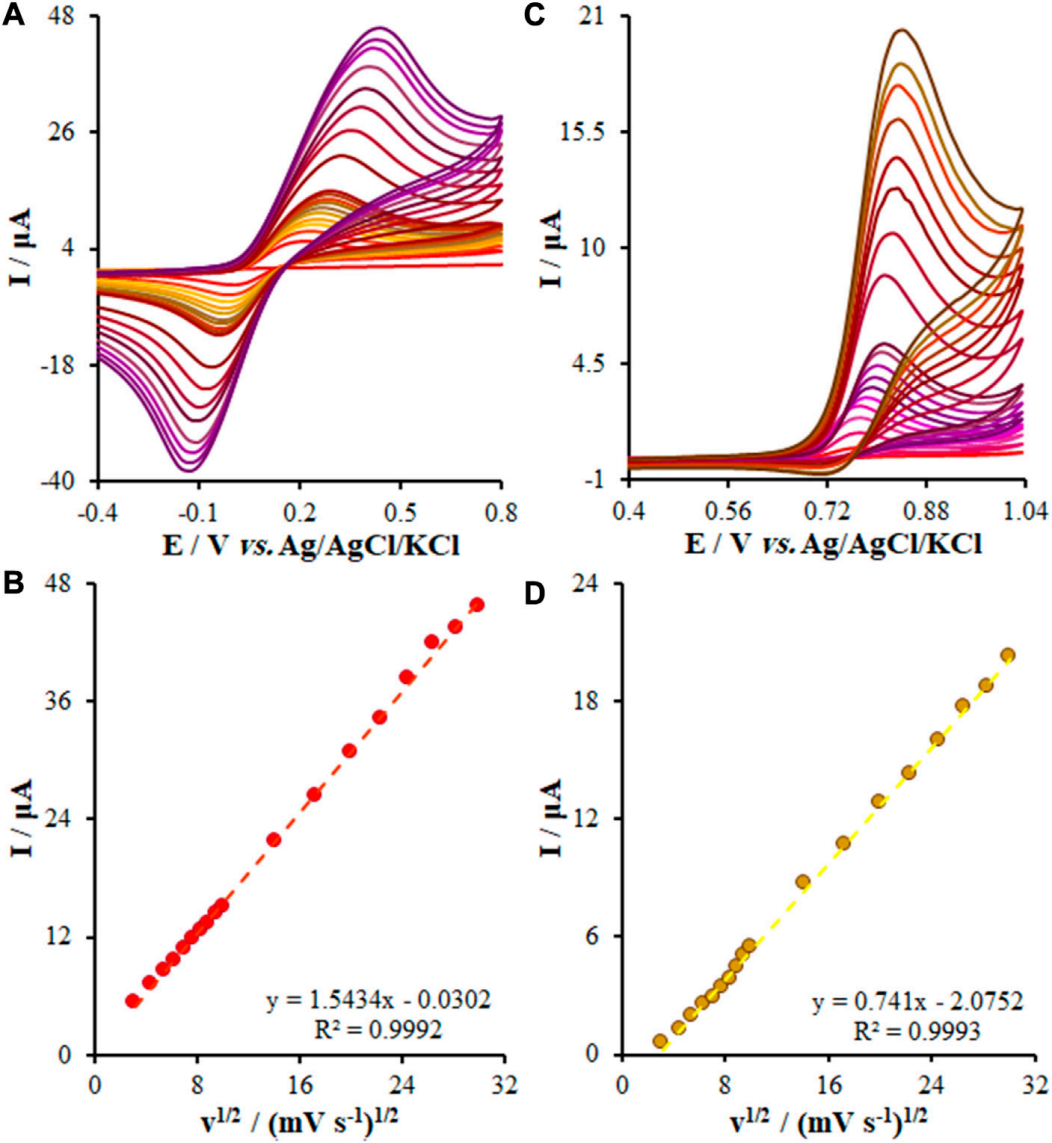
**(A,C)** CVs of FL-NS Tb^3+^/ZnO/CPE in pH 7.0 in the presence of KYN (150.0 µM) and TRP (55.0 µM) at various scan rates (from inner to outer curve): 10, 20, 30, 40, 50, 60, 70, 80, 90, 100, 200, 300, 400, 500, 600, 700, 800, and 900 mV/s. **(B,D)** The plots of peak currents vs. υ^1/2^.

It should be noted that the potential scan rate has been introduced as a major variable employed in electrochemistry for investigating the oxidation of the samples, the reaction mechanism of reduction, or obtaining the kinetic variable. In this step, we adjusted the potential scan rate of the KYN and TRP oxidation over the FL-NS Tb^3+^/ZnO/CPE surface at 10–900 mV/s but we used CV to determine its effect in 0.1 M PBS (pH = 7.0). See Figures 7A,C for more information. With the enhancement in the scanning rate, it was found that the current increased, and Figures 7B, D shows the current curve to ν^1/2^ (square of the scan rate), depicting a linear relationship based on Eqs 2, 3. As seen, the samples’ diffusion limits the oxidation reaction of KYN and TRP, respectively.

$$I_{p}=1.5434\;\nu^{1/2}-0.0302\;\left(R^{2}=0.9992\right)$$ (2)

$$I_{p}=0.741\nu^{1/2}-2.0752\left(R^{2}=0.9993\right)$$ (3)

### 3.7 Electrocatalytic oxidation of KYN and TRP using chronoamperometry

**FIGURE 8:**
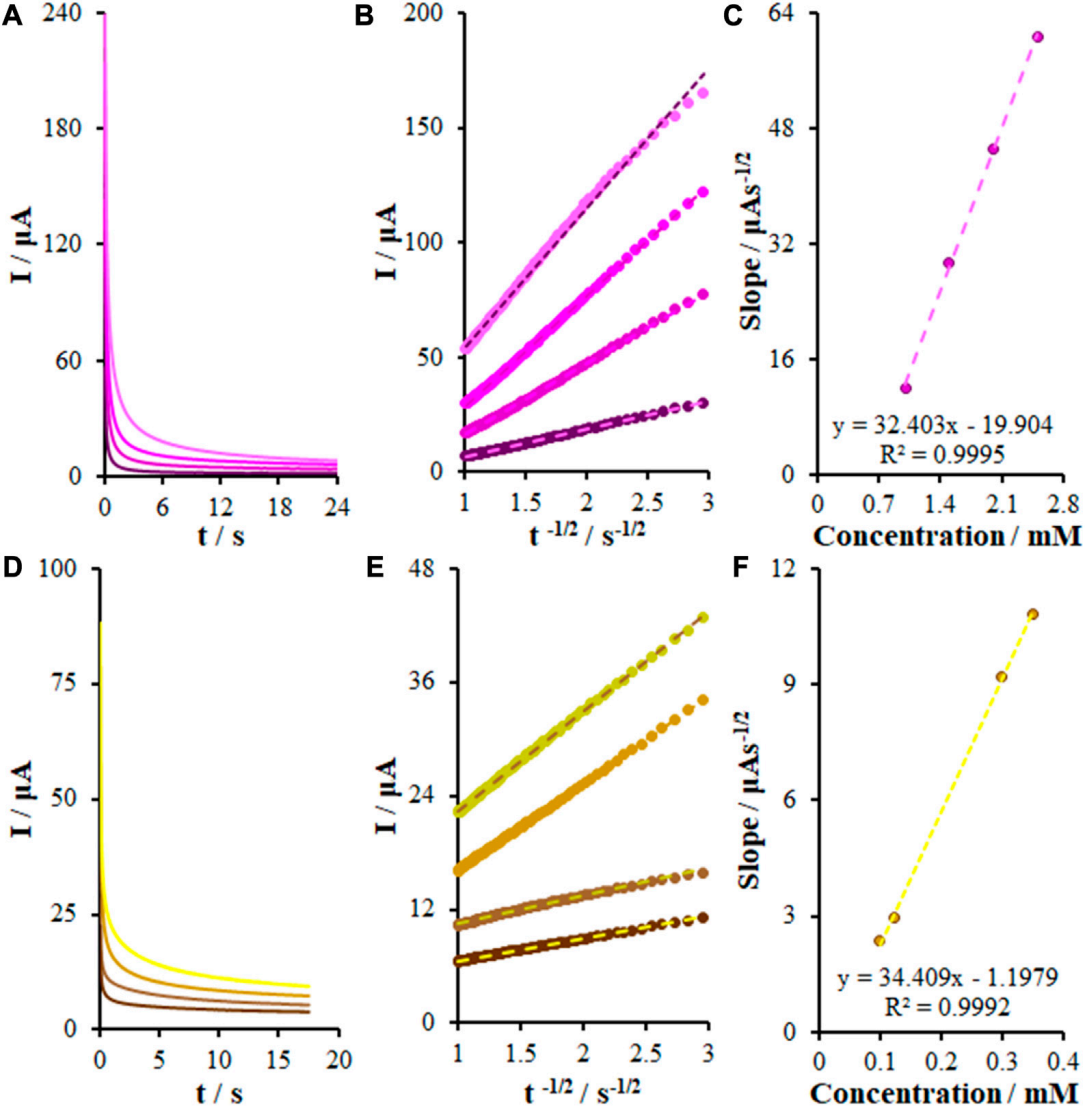
**(A)** Chronoamperograms obtained at FL-NS Tb^3+^/ZnO/CPE in 0.1 M PBS (pH 7.0) for different concentrations of KYN. The numbers 1–4 correspond to: 1.0, 1.5, 2.0, and 2.5 mM of KYN. **(B)** Plots of I vs. t^−1/2^ obtained from chronoamperograms 1–4. **(C)** Plot of the slope of the straight lines against KYN concentration. **(D)** As **(A)** for different concentrations of TRP. The numbers 1–4 correspond to: 0.1, 0.125, 0.3, and 0.35 mM of TRP. **(E)** Plots of I vs. t^−1/2^ obtained from chronoamperograms 1–4. **(F)** Plot of the slope of the straight lines against TRP concentration.

Experts in the field use chronoamperometry to measure the diffusion coefficient of electroactive samples. According to this approach, a potential is applied to the static electrode with a specific floating area in a solution with an electro-active compound at a given concentration. Then, the working electrode potential is applied to the diffusion platform of the electroactive samples, and a current–time dependence occurs. There was a relationship between the current–time curve and variations in the concentration gradient near the electrode surface. As time increased, the diffusion layer widened and inhibited the electroactive compound from approaching the surface of the electrode by decreasing the concentration slope of the profile. Hence, the current intensity in a flat electrode decreased with time (Cottrell Equation). Based on the Cottrell Equation, the emission current from the electrochemical reaction of an electroactive sample would be followed by Eq. 4 (Bard and Faulkner, 2001):

$$I=nFAD^{1/2}C_{b}\pi^{-1/2}t^{-1/2}$$ (4)

where D represents the diffusion coefficient (cm^2^/s), C refers to the concentration of the electroactive compound in terms of mol/cm^3^, and n refers to the stoichiometric number of electrons involved in the reaction (Supplementary Figure S1). In the case of plotting the flow changes with the reverse square time for a chronoamperogram, the slope equals nFACD^1/2^/π^1/2^, in which knowledge of C, F, n, and A can help to calculate the diffusion coefficient. We also examined the oxidations of KYN and TRP on the FL-NS Tb^3+^/ZnO/CPE surface through chronoamperometry (Figures 8A, D) and the current diagram in terms of t^−1/2^ was found to be linear for various concentrations (Figures 8B, E). Figures 8C, F depict the charts’ slope for diverse concentrations, with diffusion coefficients of 1.02 × 10^−6^ cm^2^/s and 1.15 × 10^−6^ cm^2^/s for KYN and TRP, respectively.

### 3.8 The analytical function of the modified electrode in detecting KYN and TRP

**FIGURE 9:**
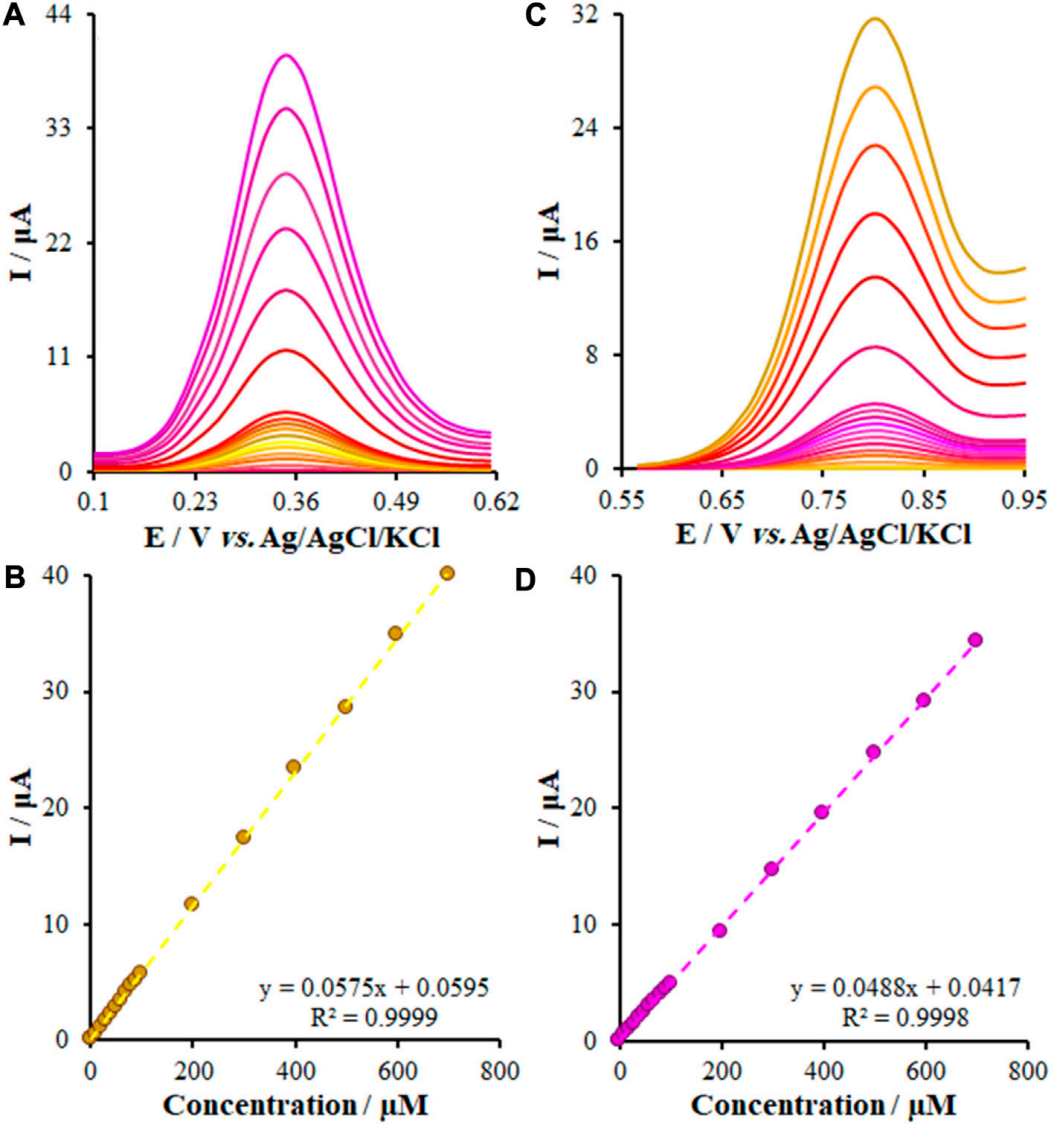
**(A,C)** DPVs of the KYN and TRY at the FL-NS Tb^3+^/ZnO/CPE in PBS (pH 7.0) at a scan rate of 50 mV s^−1^, respectively, Concentrations from inner to outer of curves: (0.001, 1.0, 10.0, 20.0, 30.0, 40.0, 50.0, 60.0, 70.0, 80.0, 90.0, 100.0, 200.0, 300.0, 400.0, 500.0, 600.0, and 700.0 μM). **(B,D)** Plots of I vs. Concentrations.

As mentioned above, we employed DPV for the evaluation and determination of small concentrations of KYN and TRP. Figure 9 represents the DP voltammograms of the FL-NS Tb^3+^/ZnO/CPE surface in the optimum conditions that ranged from 0.001 to 700.0 μM. Eqs 5, 6 give the linear relationship of I_pa_ and the concentrations:

$$I\;\left(\mu A\right)=0.0575C_{\text{KYN}}\;\left(\mu M\right)+0.0595\;\left(R^{2}=0.9999\right)$$ (5)

$$I\;\left(\mu A\right)=0.0488\;C_{\text{TRP}}\;\left(\mu M\right)+0.0417\;\left(R^{2}=0.9998\right)$$ (6)

The limit of detection (LOD) of this new approach, obtained from Eq. LOD = 3S_b_/M [so that M represents the slope of the calibration curve, S_b_ stands for the blank standard deviation (SD)], was found to be 0.34 nM and 0.22 nM for KYN and TRP, respectively. Hence, this performance and greater sensitivity resulted from the synergistic effect of FL-NS Tb^3+^ and ZnO, increasing the transport rate of electrons and detecting the limited levels of KYN and TRP.

### 3.9 Selectivity of the modified electrode in relation to KYN and TRP

In this step, we determined the impact of numerous interference samples (α) in the real samples (Table 2) on the potential and peak current of the DP voltammograms for investigating the FL-NS Tb^3+^/ZnO/CPE selectivity to KYN and TRP. Then, the DPV of 50.0 μM KYN and TRP were recorded five times in the presence of α with 100 times the concentration in comparison with KYN and TRP. The next step addressed the comparison of the average of the peaks E_p_ and I_P_ with the KYN and TRP peaks E_p_ and I_P_ in the absence of α. In the case of ˂5% E_p_ and I_P_ in the presence of α compared to its absence, the interference samples and the above stages continued to reach a 5% difference. The interference samples were chosen according to the common molecules and ions in the drug samples and biological fluids. The α samples were not interfered with when measuring KYN and TRP, revealing the very good selectivity of FL-NS Tb^3+^/ZnO/CPE to both drugs and their probable measurement in the real samples.

**TABLE 2:** The effect of interferences on the measurement of 50.0 µM of KYN and TRP with FL-NS Tb^3+^/ZnO/CPE in buffer phosphate 0.1 M with pH = 7.0.

Species	The molar ratio of interference species, α, to the concentration of KYN and TRP
Na^+^, K^+^, Mg^2+^, Ca^2^	1,000
NO_3_^−^, CO_3_^2–^, Cl^−^	1,000
Ascorbic acid, Oxalate, Glycine, Fructose, Sucrose, Glucose, Lactose	800
Uric acid, Dopamine, Acetylsalicylic acid	700

### 3.10 Simultaneous detection of KYN and TRP

**FIGURE 10:**
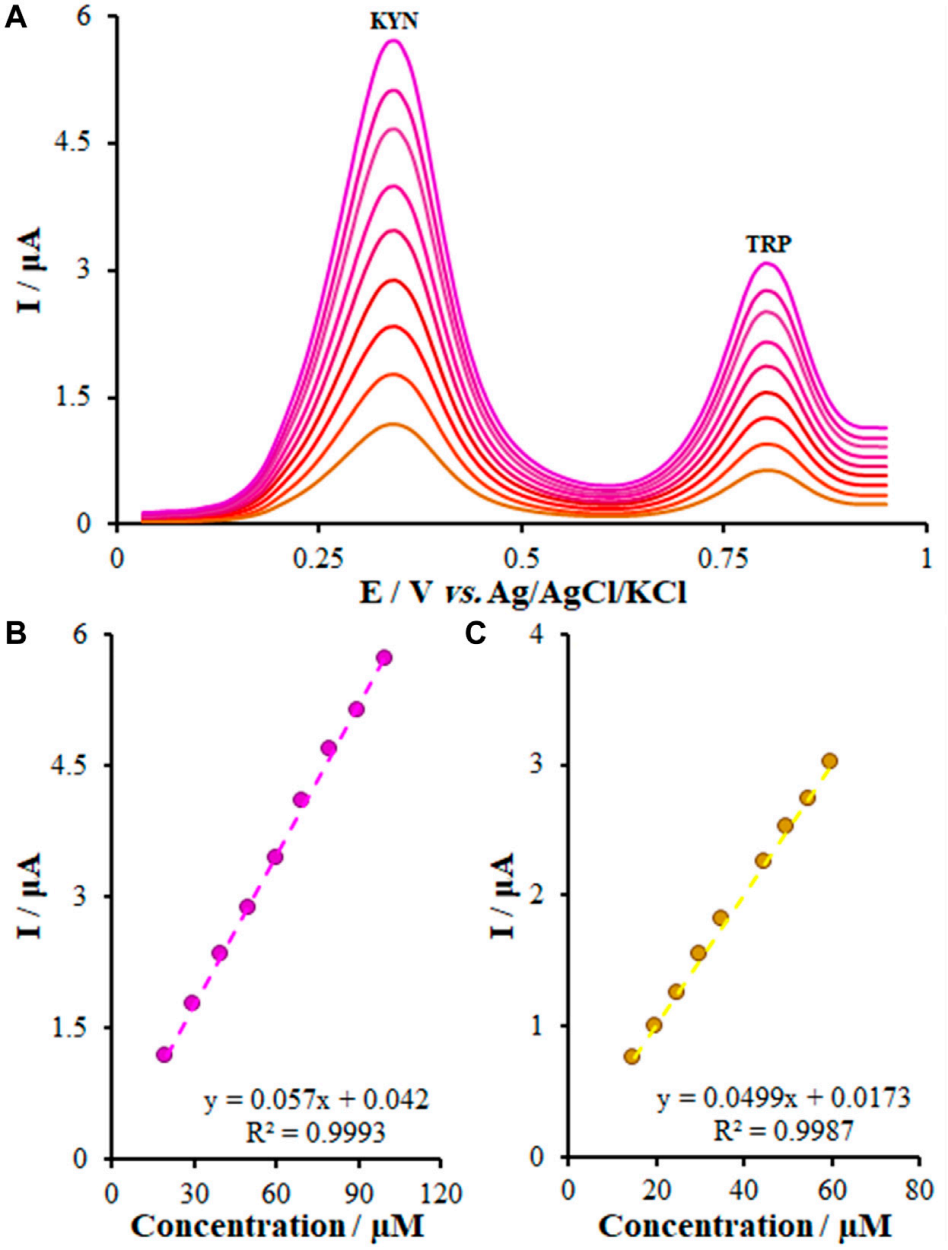
**(A)** DPVs of FL-NS Tb^3+^/ZnO/CPE in 0.1 M PBS (pH 7.0) containing different concentrations of KYN and TRP (from inner to outer) mixed solutions of Eq. 1 20.0 + 15.0; (2) 30.0 + 20.0; (3) 40.0–25.0; (4) 50.0–30.0; (5) 60.0 + 35.0; (6) 70.0 + 45.0; (7) 80.0 + 50.0; (8) 90.0 + 55.0; and (9) 100 + 60.0 μM KYN and TRP, respectively, **(B)** Plot of the peak currents as a function of KYN concentration, and **(C)** Plot of the peak currents as a function of TRP concentration.

It is widely accepted that simultaneous measurements of the medicines using a modern technique by a new electrode could exhibit acceptable functions in real samples. Hence, we assessed the function of the FL-NS Tb^3+^/ZnO/CPE electrode in simultaneous measurement of KYN and TRP (Figure 10A). With regard to Figures 10B, C, concentration of KYN and TRP were changed from 20.0-100.0 μM for KYN and 15.0-60.0 for TRP. As shown by Figure 10A, no changes were observed in the potential of KYN and TRP and peak current in the presence of each of them. Thus, an FL-NS Tb^3+^/ZnO/CPE electrode could be used for measuring samples of both KYN and TRP without interfering with each other.

### 3.11 Reproducibility and stability

To examine the reproducibility of the preparation process of the electrode, we conducted measurements in 50.0 μM solutions of KYN and TRP using five modified electrodes with the same DPV and the value of the relative standard deviation (RSD) of the anodic peak current (mean of 3 measurements/electrode) equaled 2.84 and 2.52 (Supplementary Figure S1). The findings indicated the acceptable reproducibility of the modified electrode in the voltammetric measurements and electrode production. Then, we put the electrode into a buffer medium at room temperature for 4 weeks and analyzed 50.0 μM solutions of KYN and TRP to evaluate the modified electrode stability. Ultimately, the peak currents (3.12% and 2.26%) declined, demonstrating the acceptable stability of the prepared electrode (Supplementary Figure S2).

### 3.12 Comparison of our method with others in the literature

The comparison of analytical efficacy between the as-fabricated electrode and other electrochemical and non-electrochemical methods was performed for KYN and TRP (Table 3). As shown in Table 3, the detection limit and linear range of the as-fabricated sensor were better than electrochemical and non-electrochemical methods (Zhao et al., 2010; Wu et al., 2023; Kambale and Lokhande, 2023; Zhao et al., 2023; Taherizadeh et al., 2023; Guo et al., 2023). When comparing chromatography methods with electrochemical methods, these methods are expensive, sophisticated, and multi-process techniques, with the need for sample preparation, pre-filtration, extraction, and temperature monitoring. The as-fabricated sensor is potentially able to determine the trace amounts of KYN and TRP in various media. In addition, it is noteworthy that the voltammetry method determines KYN and does not utilize simultaneous determination with TRP. As seen in Table 3, the as-fabricated electrode for electrochemically sensing KYN and TRP generally showed admirable properties for measurement speed, sensitivity, detection limit, and linear range when compared to other methods reported in the literature.

**TABLE 3:** Performance comparison of FL-NS Tb^3+^/ZnO/CPE for the simultaneous determination of KYN and TRP.

Method	Modifier	Linear range (µM)	Detection limit	Ref.
KYN				
High-performance liquid chromatography	-	1.0–10.0 µM	0.03 µM	Zhao et al. (2010)
High-performance liquid chromatography	-	0.02–1.0 (µg/mL)	6.0 (ng/mL)	Du et al. (2018)
Adsorptive stripping Voltammetry	-	2.5 nM-250.0 µM	1.72 nM	Furlanetto et al. (1997)
Voltammetry	Metal-organic frameworks, MIL-101(Cr)	0.1–150.0	17.0 nM	Bornaei et al. (2023)
Voltammetry	FL-NS Tb^3+^/ZnO	0.001–700.0	0.34 nM	This work
**TRP**				
High-performance liquid chromatography	-	10.0–100.0 µM	0.4 µM	Zhao et al. (2010)
High-performance liquid chromatography	-	0.2–20.0 (µg/mL)	5.0 (ng/mL)	Du et al. (2018)
Voltammetry	Zinc oxide nanoparticles	10.0–40.0 µM	0.57 µM	Shruthi Vishwanath et al. (2023)
Voltammetry	Poly (9-aminoacridine)	5.0–200.0 µM	0.035 µM	Yang et al. (2022)
Voltammetry	FL-NS Tb^3+^/ZnO	0.001–700.0	0.22 nM	This work

### 3.13 Measurement of real samples

The new electrode was employed for determining KYN and TRP in blood serum and urine samples to evaluate the capability of FL-NS Tb^3+^/ZnO/CPE for oxidation of the analytes. The standard addition procedure was applied for the measurements because of the complexity of the real samples’ matrices. Based on the findings, the recovery percentage for the blood serum samples ranged from 97.7%–101.2% and for the urine samples from 98.7%–103.6% (Table 4). The acceptable percentage of recovery and RSD values of ˂5% demonstrate the high functionality of the FL-NS Tb^3+^/ZnO/CPE to measure KYN and TRP in samples with real matrices.

**TABLE 4:** Determination of KYN and TRP in real samples. All the concentrations are in μM (*n* = 5).

Sample	Spiked		Found		Recovery (%)		R.S.D. (%)	
	KYN	TRP	KYN	TRP	KYN	TRP	KYN	TRP
Human blood serum	0	0	-	-	-	-	-	-
	8.0	6.0	7.9	6.2	98.7	103.6	1.7	2.6
	16.0	12.0	16.2	12.1	101.2	100.8	2.3	2.8
Urine	0	0	-	-	-	-	-	-
	20.0	15.0	20.2	14.8	101.0	98.7	3.1	1.8
	40.0	30.0	39.1	30.1	97.75	100.3	2.9	2.1

Legends for the Figures.

## 4 Conclusion

We employed a hydrothermal method to provide FL-NS Tb^3+^/ZnO and thus FL-NS Tb^3+^/ZnO was employed as a CPE modifier for sensing KYN and TRP through DVP and CV. The major merits of the mentioned method were its rapid and simple simultaneous determination of KYN and TRP. In addition, we showed the facilitation of the transition of KYN and TRP targets into the boundary of the electrolyte/electrode by FL-NS Tb^3+^/ZnO. Moreover, electron transportation was facilitated by the conductivity and porous structure of FL-NS Tb^3+^/ZnO. According to the results, the peak potential of KYN and TRP oxidation was 0.49 V and 0.37 V in FL-NS Tb^3+^/ZnO/CPE, respectively. Furthermore, the modified electrodes could probably be used for quantitative analysis of KYN and TRP in the plasma sample of humans, resulting in acceptable outcomes. Therefore, measuring KYN and TRP with the help of FL-NS Tb^3+^/ZnO/CPE can be considered a modern method. Hence, this new sensor could be utilized as a model for modifying other existing sensors.

## Data Availability

The original contributions presented in the study are included in the article/Supplementary Material, further inquiries can be directed to the corresponding author.
