# Supplementary material for: Electrochemical detection of kynurenic acid in the presence of tryptophan with the carbon paste electrode modified with the flower-like nanostructures of zinc oxide doped with terbium
Source: Front Chem. 2023 Sep 22;11:1250994. doi: 10.3389/fchem.2023.1250994 (PMC10556251; doi:10.3389/fchem.2023.1250994)
Supplement: Supplementary file 1 [file DataSheet1.docx]

**Electrochemical Detection of kynurenic acid in the Presence of tryptophan with the Carbon Paste Electrode modified with the Flower-like Nanostructures of Zinc Oxide Doped with Terbium**

**
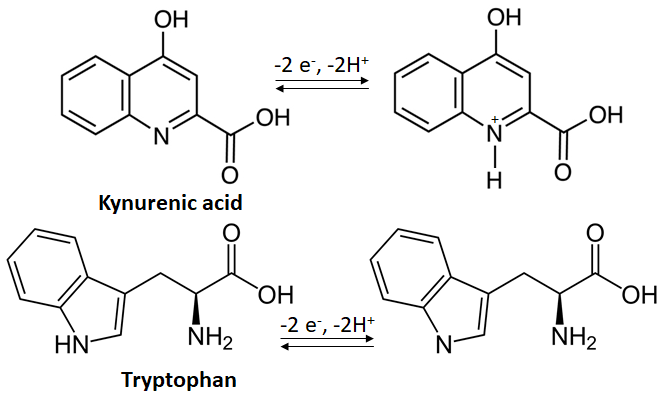
**

**Figure S1.** The suggested oxidation mechanism of KYN and TRP at FL-NS Tb^3+^/ZnO/CPE.

**
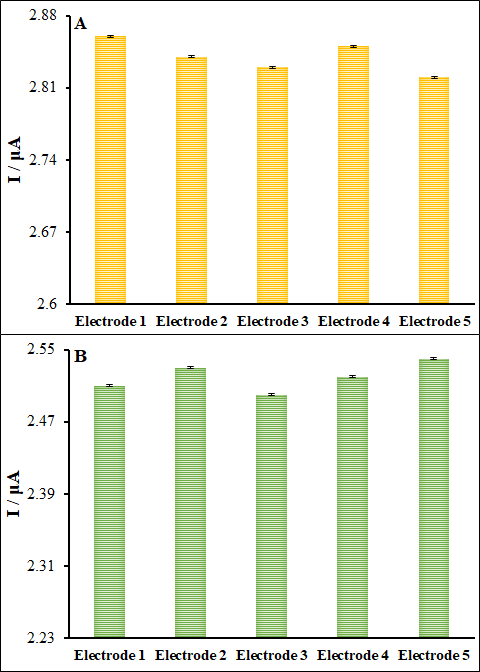
**

**Figure S2.** Current responses of five FL-NS Tb^3+^/ZnO/CPE fabricated under the same conditions.


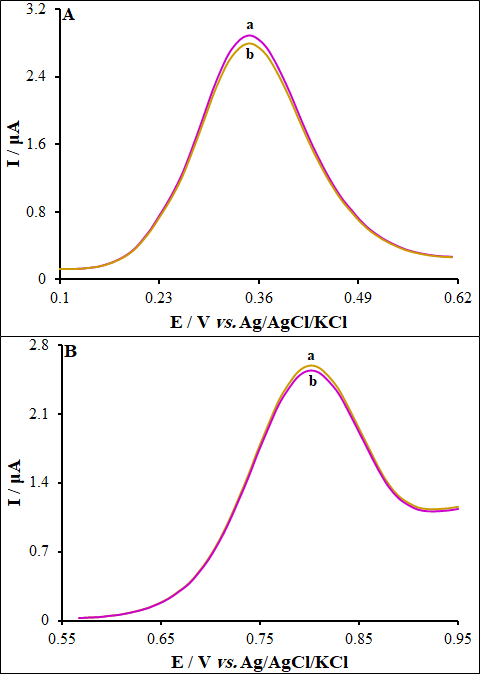


**Figure S3.** (A) DPVs of modified electrode (a) containing 50.0 μM of KYN and (b) after 4 weeks. (B) DPVs of modified electrode (a) containing 50.0 μM of TRP and (b) after 4 weeks.
